# Quantum-inspired analysis of neural network vulnerabilities: the role of conjugate variables in system attacks

**DOI:** 10.1093/nsr/nwae141

**Published:** 2024-04-11

**Authors:** Jun-Jie Zhang, Deyu Meng

**Affiliations:** Division of Computational physics and Intelligent modeling, Northwest Institute of Nuclear Technology, Xi’an 710024, China; School of Mathematics and Statistics and Ministry of Education Key Lab of Intelligent Networks and Network Security, Xi’an Jiaotong University, Xi’an 710049, China; Pazhou Lab, Guangzhou 510335, China

**Keywords:** neural network, adversarial attack, accuracy-robustness trade-off, uncertainty principle, quantum physics

## Abstract

Neural networks demonstrate vulnerability to small, non-random perturbations, emerging as adversarial attacks. Such attacks, born from the gradient of the loss function relative to the input, are discerned as input conjugates, revealing a systemic fragility within the network structure. Intriguingly, a mathematical congruence manifests between this mechanism and the quantum physics’ uncertainty principle, casting light on a hitherto unanticipated interdisciplinarity. This inherent susceptibility within neural network systems is generally intrinsic, highlighting not only the innate vulnerability of these networks, but also suggesting potential advancements in the interdisciplinary area for understanding these black-box networks.

## Teaser

The study draws an unexpected parallel between quantum physics and neural networks, uncovering a fundamental uncertainty principle that highlights the intrinsic vulnerabilities of AI systems.

## INTRODUCTION

Despite the widely demonstrated success across various domains—from image classification [1] and speech recognition [2] to predicting protein structures [3], playing chess [4] and other games [5], etc.—deep neural networks have recently come under scrutiny for an intriguing vulnerability [6,7]. The robustness of these intricately trained models is being called into question, as they seem to falter under attacks that are virtually imperceptible to human senses.

A growing body of both empirical [8–16] and theoretical [17–20] evidence suggests that these sophisticated networks can be tripped up by minor, non-random perturbations, producing high-confidence yet erroneous predictions—a striking and quite succinct example being the fast gradient sign method (FGSM) attack [18]. These findings raise significant concerns about the vulnerabilities of such neural networks. If their performance can indeed be undermined by such slight disruptions, the reliability of technologies that hinge on state-of-the-art deep learning could potentially be at risk.

A natural question emerges concerning the vulnerability of deep neural networks. Despite the classical approximation theorems [21–24] promising that a neural network can approximate a continuous function to any desired level of accuracy, is the observed trade-off between accuracy and robustness an intrinsic and universal property of these networks?

This query stems from the intuition that stable problems, described by stable functions, should intrinsically produce stable solutions. The debate within the scientific community is still ongoing. If this trade-off is indeed an inherent feature then a comprehensive exploration into the foundations of deep learning is warranted. Alternatively, if this phenomenon is merely an outcome of approaches to constructing and training neural networks, it would be beneficial to concentrate on enhancing these processes, as has already been undertaken, e.g. the certified adversarial robustness via randomized smoothing [25–27] and the concurrent training strategy [19,28–34].

In this study, we uncover an intrinsic characteristic of neural networks: their vulnerability shares a mathematical equivalence with the uncertainty principle in quantum physics [35,36]. This is observed when gradient-based attacks [18,37–41] on the inputs are identified as conjugate variables, in relation to these inputs. Since modern network structures always include a loss function with respect to some inputs, we are allowed to ‘design’ the conjugate variables by taking the gradient of the loss function with respect to the input variables. These conjugate variables, when involved in the inputs, can drastically decrease the prediction accuracy. Thus, the uncertainty principle is a natural result of minimizing the loss function, which is inevitable and universal.

Taking into account a trained neural network model, denoted *f*(*X*, θ), where θ signifies the parameters and *X* represents the input variable of the network, we observe a consistent pattern. The network cannot achieve arbitrary levels of measuring certainties on two factors simultaneously: the conjugate variable ∇_*X*_*l*(*f*(*X*, θ), *Y*) (where *Y* denotes the underlying ground-truth label of *X*) and the input *X*, leading to the observed accuracy-robustness trade-off. This phenomenon, similar to the quantum physics’ uncertainty principle, offers a nuanced understanding of the limitations inherent in neural networks.

## RESULTS

### Conjugate variables as attacks

In quantum mechanics, the concept of conjugate variables plays a critical role in understanding the fundamentals of particle behavior. Conjugate variables are a pair of observables, typically represented by operators, which do not commute. This non-commutativity implies that the order of their operations is significant and it is intrinsically tied to Heisenberg’s uncertainty principle [35,36]. A prime example of such a pair is the position operator, $\hat{x}_{\text qt}$, and the momentum operator, $\hat{p}_{\text qt}=-i{\partial }/{\partial x_{\text qt}}$. Here, the order of operations matters such that $\hat{x}_{\text qt}\hat{p}_{\text qt}$ is not equal to $\hat{p}_{\text qt}\hat{x}_{\text qt}$, indicating the impossibility of simultaneously determining the precise values of both the position and momentum. This inherent uncertainty is quantitatively expressed in Heisenberg’s uncertainty relation: $\Delta x_{\text qt} \Delta p_{\text qt} \ge \frac{1}{2}$ with Δ*x*_qt_ and Δ*p*_qt_ respectively representing the standard deviations of the position and momentum measurements.

Drawing an analogy from quantum mechanics, we can formulate the concepts of conjugate variables within the realm of neural networks. Specifically, the features of the input data provided to a neural network can be conceptualized as feature operators, denoted $\hat{x}_{i}$, while the gradients of the loss function with respect to these inputs can be viewed as attack operators, denoted $\hat{p}_{i}={\partial }/{\partial x_{i}}$. Here, subscript *i* refers to the *i*th feature of the entire input feature vector. The attack operators, corresponding to the gradients on inputs, hold a clear relationship with gradient-based attacks, such as the FGSM attack (the application of such attacks often involves a sign function, although this is not strictly necessary [42,43]).

This analogy leads us to an inherent uncertainty relation for neural networks, mirroring Heisenberg’s uncertainty principle in quantum mechanics. Providing a trained neural network with properly normalized loss functions, the relation reads $\Delta x_{i} \Delta p_{i} \ge \frac{1}{2}$ (see the derivations in the online supplementary material). This relation, relying on both the dataset and the network structure, suggests that there exists an intrinsic limitation in precisely measuring both features and attacks simultaneously. This intrinsically reveals an inherent vulnerability of neural networks, echoing the uncertainty we observe in the quantum world.

To intuitively visualize the manifestation of Δ*x* = (∑Δ*x_i_*)^1/2^ and Δ*p* = (∑Δ*p_i_*)^1/2^ within neural networks, we use the MNIST dataset as a representative example. The neural network is trained and subsequently subjected to attacks at each training epoch.

**Figure 1.**
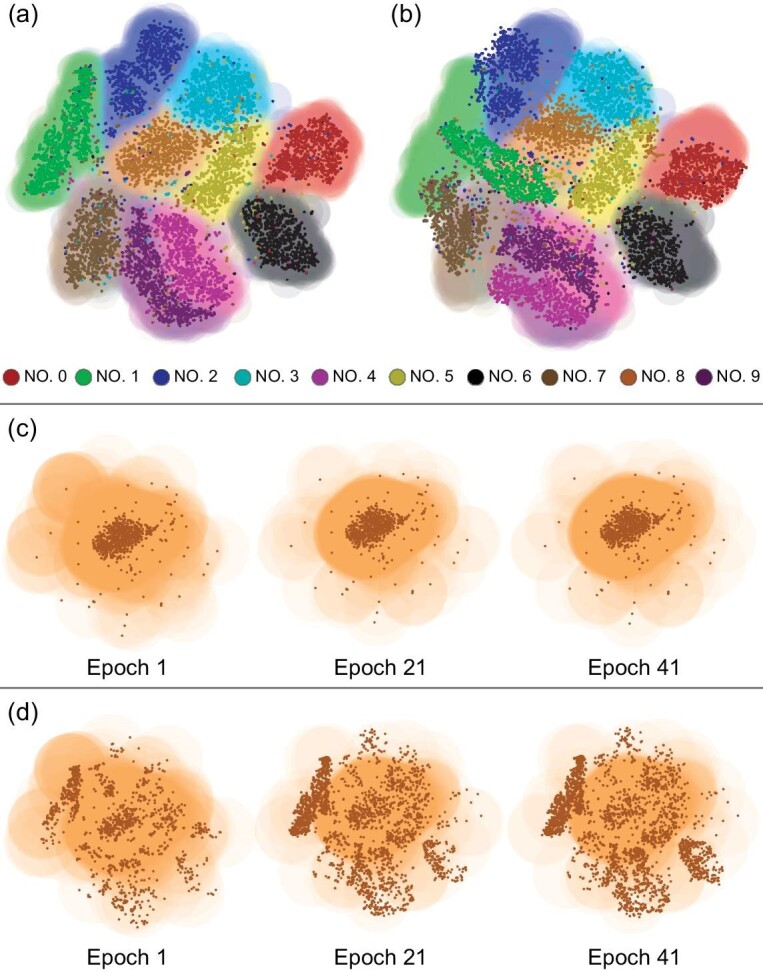
Illustration of Δ*x* and Δ*p* in a three-layer convolutional neural network trained on the MNIST dataset over 50 epochs. The data’s high-dimensional feature space was reduced to two dimensions using the t-distributed stochastic neighbor embedding (t-SNE) algorithm for easy visualization. (a) Shaded regions indicate the class predictions obtained by the finally trained network, and the colors imposed on individual points indicate the true labels of corresponding test samples. (b) All test samples were subjected to the projected gradient descent (PDG) adversarial attack method [37,38] with ϵ = 0.1 and α = 0.1/4 over four iterative steps. We see that these adversarially perturbed samples evidently deviate from the class regions they should be located within. (c) The prediction region evolution for the digit ‘8’ is displayed at epochs 1, 21 and 41. The deeper the color, the more confident the prediction by the network. (d) The shaded area is similar to (c), but with points representing the adversarial predictions of the attacked images, illustrating the temporal impact of the PDG attack on model accuracy.

In this scenario, a trained network partitions the hyperspace (the space inhabited by the samples) into distinct regions. A given input, represented as a point in this space, is classified based on the label of the region it falls within. After 50 epochs of training, the shaded areas encapsulate most correctly labeled data points (Fig. 1a). Conversely, the attacks shift these input points slightly, leading to misclassification. The shifted points do not overlap with the regions defined by the trained network (Fig. 1b).

We pay particular attention to class number 8, which exhibits the most interconnections with other classes. This class is further illustrated in Fig. 1c and d. As the training epochs progress, the ‘effective radius’ of the shaded area shrinks, causing the area to gradually coincide with the correctly labeled data points (Fig. 1c). Simultaneously, the ‘effective radius’ of the attacked points begins to deviate further from the shaded regions, and thus from the correctly labeled data (Fig. 1d). An intuitive correspondence of the ‘effective radii’ are the domain representations in Fourier transformation, where a narrow time domain representation (Δ*t*) corresponds to a wider frequency domain representation (Δω), restricted by a similar relation, Δ*t*Δω ≥ 1/4π. Intuitively, we may think of Δ*t* and Δω as correspondences to the ‘effective radius’ in neural networks.

This visualization reveals an inherent trade-off: a reduction in the effective radius of the trained class corresponds to an increase in the effective radius of the attacked points. These two radii can be conceptualized as the visual representations of the uncertainties, Δ*x* and Δ*p*, highlighting the delicate balance between precision and vulnerability in neural networks.

In addition to the adversarial attacks explored in this study, there exist analogous effective conjugates in other types of adversarial attacks as well [37,39–41]. While we are currently unable to explicitly define the conjugates associated with black-box attacks as referenced in [44,45], it is plausible that these methods may adhere to the same underlying principle.

### Manifestation of the uncertainty principle in neural networks

**Figure 2.**
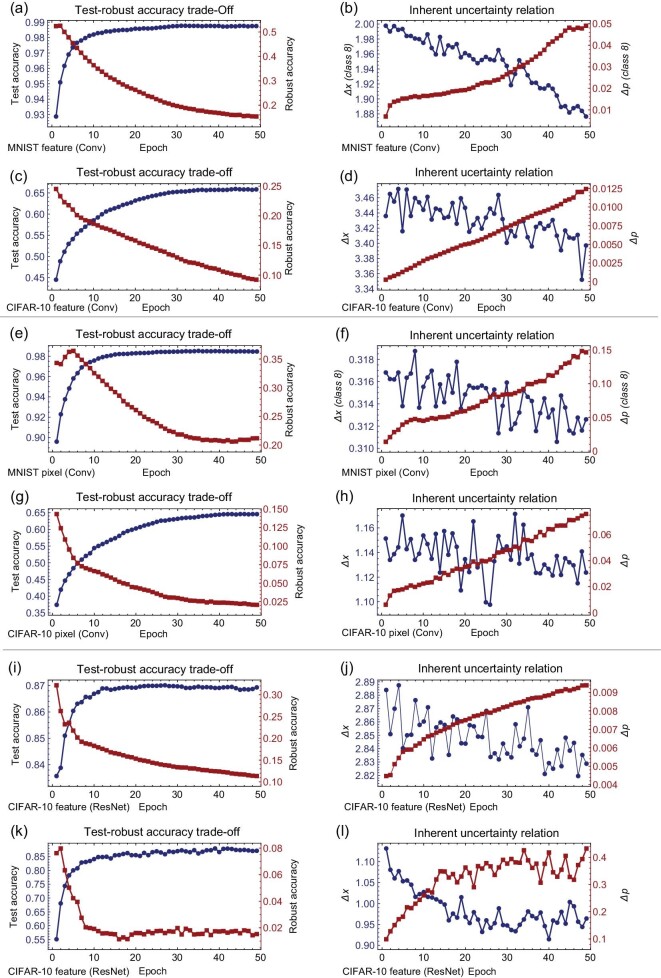
Results of the three different types of neural networks: a three-layer convolutional network running on the MNIST dataset, a four-layer convolutional network on the CIFAR-10 dataset and a residual network [46] with eight convolutional layers on the CIFAR-10 dataset. The term ‘feature’ in the labels represents the results obtained by attacking the features of the input images, while ‘pixel’ corresponds to attacks directed at the pixels themselves. Each neural network underwent training for a span of 50 epochs. The quantities Δ*x* and Δ*p* were determined through high-dimensional Monte Carlo integrations. Subfigures (a), (c), (e), (g), (i) and (k) depict the test and robust accuracy metrics, with the robust accuracy evaluated on images perturbed by the PDG adversarial attack method, using parameters ϵ = 8/255 and α = 2/255 across four iterative steps. Subfigures (b), (d), (f), (h), (j) and (l) illustrate the trade-off relationship between Δ*x* and Δ*p*.

The shaded areas in Fig. 1a are actually representative of wave functions in quantum physics. Specifically, for the MNIST dataset, we have 10 corresponding wave functions, corresponding to 10-digit number classes. Therefore, the uncertainty relation $\Delta x \Delta p \ge \frac{1}{2}$ shown in Fig. 1c and d should be reinterpreted as $\Delta x[\text{class 8}] \Delta p[\text{class 8}] \ge \frac{1}{2}$, indicating that we are concentrating on the class of number 8. This equation is a clear depiction of the trade-off between Δ*x*[class8] and Δ*p*[class8], as depicted in Fig. 2b, accompanied by the associated trade-off between accuracy and robustness (Fig. 2a). The CIFAR-10 dataset, having a higher complexity than MNIST, poses a potential indeterminacy in identifying a specific class that has more connectivity with other classes. In this case, the average values Δ*x* = Mean(Δ*x*[Allclasses]) and Δ*p* = Mean(Δ*p*[Allclasses]) are employed instead. Similar results obtained on CIFAR-10 underscore the inherent uncertainty relation that drives the accuracy-robustness trade-off, as demonstrated in Fig. 2c and d.

## DISCUSSIONS

To elucidate the impacts of the uncertainty principle across various strata, we examine its repercussions on input selection, variabilities in network architectures and the interplay between physical sciences and artificial intelligence as follows.

### Attacking features is more effective than attacking pixels

The pixels in our dataset serve as the raw, unprocessed data, gathered directly from the detectors. These pixels carry the features that serve as an accurate representation of the real world. While there is a possibility of manipulating these features, it is more common and practical to focus on the pixels themselves. By doing so, we can observe the accuracy-robustness trade-off (Fig. 2e and g), a fundamental concept that is underpinned by the uncertainty relation, as seen in Fig. 2f and h.

However, it is important to note, as evidenced by the testing accuracy results from the MNIST dataset, that there is an initial learning curve or ‘kick’ that is encountered (Fig. 2e). This is to be expected as the neural network must first familiarize itself with, or ‘learn,’ the features before it can effectively classify the images.

While processing the initial learning stages, it is also worth noting the fluctuation in both Δ*x* and Δ*p* for input pixels. This fluctuation is more pronounced than that seen in the features, highlighting the random exploration nature of the learning algorithm. As illustrated in Fig. 2h, these fluctuations could be attributed to the inherent randomness of the learning process, a factor that is crucial to potentially uncover more optimal weight configurations.

Meanwhile, since attacking features are more effective than attacking the pixels, we may expect that if sufficient training data are provided to the neural network, the attack will be more effective.

### Phenomenon in attacking well-designed neural networks

Typically, network structures are scrupulously architected to fit the demands of specific tasks. Take panels c, d, g and h of Fig. 2 as examples. In the figure, the network only achieves a test accuracy of around 65% due to the relatively simple network architecture. To address this, we introduce a more advanced network structure that incorporates residual networks and additional convolutional layers. This refined structure increases the accuracy to nearly 90%. (Given that the quantities Δ*x* and Δ*p* are approximately computed through high-dimensional Monte Carlo integrations, a process that is exceedingly time consuming, we can only feasibly perform these computations for the network with such complexity. If they could be calculated more accurately under more complex and accurate networks with stronger computational resources, we believe that the calculated patterns will better conform to the expected regularities.) One can still observe a clear pattern in the trade-off between Δ*x* and Δ*p* for both features and pixels (Fig. 2i–l). Besides, this trade-off is also more pronounced for features than for pixels. Understanding this trade-off allows for a more effective optimization of the network structure. In closing, constructing a network structure that best fits the task at hand is pivotal in delivering optimal performance.

### Neural network as a complex physical system

As scientific research and engineering become increasingly reliant on artificial intelligence (AI) methods, questions about the future role of human beings in these fields naturally arise. Whether guiding AI or being guided by it, understanding the fundamental principles underpinning these sophisticated structures is paramount. One approach to glean this understanding is to treat neural networks as complex physical systems, thereby applying principles of physics to elucidate the inner mechanisms of AI.

In the study at hand, it is posited that neural networks, much like quantum systems, are subject to a form of the uncertainty principle. This connection potentially uncovers intrinsic vulnerabilities within the neural networks. A comparison of formulas from these distinct fields is presented in Table 1. Here, concepts from quantum physics such as the position, momentum and wave function are juxtaposed with their counterparts in neural networks: image, attack, normalized loss function and so on. This comparison not only reveals striking similarities, but also indicates that the methodologies employed in physical sciences could potentially be harnessed to investigate the properties of neural networks.

**Table 1.** Comparison of the uncertainty principle between quantum physics and neural networks. Subscript *i* represents the *i*th dimension. For physics, *i* stands for the spatial coordinates (*x, y* and *z*), whereas in the context of neural networks, *i* refers to the *i*th feature. When we consider pixels, *i* simply pertains to the *i*th pixel. Additionally, we utilize Dirac notation, for instance, $\langle \hat{x}_{i,\text{qt}}\rangle = \int \psi ^{*}(X)x_{i,\text{qt}}\psi (X)dX\!$, where $\langle \hat{x}_{i,\text{qt}}\rangle$ is the expectation value of the *i*th dimension. Similarly, $\langle \hat{x}_{i}\rangle = \int \psi _{Y}(X)x_{i}\psi _{Y}(X)dX$ for neural networks.

Quantum physics		Neural networks	
Position	*X* = (*x, y, z*)	*X* = (*x*_1_, …, *x_i_*, …, *x_M_*)	Image/feature (input)
Momentum (conjugate of position)	*P* = (*p_x_, p_y_, p_z_*)	*P* = (*p*_1_, …, *p_i_*, …, *p_M_*)	Attack (conjugate of input)
Wave function	ψ(*X*)	ψ_*Y*_(*X*)	Normalized loss function (neural packet)
Normalized condition	∫|ψ(*X*)|^2^*dX* = 1	∫|ψ_*Y*_(*X*)|^2^*dX* = 1	Normalized condition
Position operator	$\hat{x}_{i,\text{qt}}\psi (X)=x_{i,\text{qt}}\psi (X)$	$\hat{x}_{i}\psi _{Y}(X)=x_{i}\psi _{Y}(X)$	Feature operator
Momentum operator	$\hat{p}_{i,\text{qt}}\psi (X)=-i{\partial \psi (X)}/{\partial x_{i,\text{qt}}}$	$\hat{p}_{i}\psi _{Y}(X)={\partial \psi _{Y}(X)}/{\partial x_{i}}$	Attack operator
Standard deviation for measuring position	$\sigma _{x_{i,\text{qt}}}=\langle (\hat{x}_{i,\text{qt}}-\langle \hat{x}_{i,\text{qt}} \rangle )^{2}\rangle ^{1/2}$	$\Delta {x_{i}}=\langle (\hat{x}_{i} -\langle \hat{x}_{i}\rangle )^{2}\rangle ^{1/2}$	Standard deviation for resolving pixel
Standard deviation for measuring momentum	$\sigma _{p_{i,\text{qt}}}=\langle (\hat{p}_{i,\text{qt}}-\langle \hat{p}_{i,\text{qt}} \rangle )^{2}\rangle ^{1/2}$	$\Delta {p_{i}}=\langle (\hat{p}_{i}-\langle \hat{p}_{i}\rangle )^{2}\rangle ^{1/2}$	Standard deviation for resolving attack
Uncertainty relation	$\sigma _{x_{i,\text{qt}}}\sigma _{p_{i,\text{qt}}}\ge \frac{1}{2}$	$\Delta {x_{i}}\Delta {p_{i}}\ge \frac{1}{2}$	Uncertainty relation

Meanwhile, the inherent uncertainty principle should also hold for large fundamental models in principle. Since these large networks have much more complex structures and are trained with a huge amount of data, they can be treated as complex physical systems containing various features at different levels and scales. Therefore, these large models should be more sensitive to the gradient-based conjugates. In fact, some recent research has revealed the fact that large language models can be easily cheated by adversarial attacks [47] and jailbreak prompts [48], leading to potential risks as we rely much more on these large models. However, more thorough and versatile empirical studies along this line have yet been widely investigated, leaving a large space for future studies.

The intersection of AI and physics has the potential to provide novel insights into the intricate complexities of neural networks. For instance, the emergent capabilities exhibited by large language models might be correlated with principles found in statistical physics. Moreover, phenomena such as small data learning could be linked to concepts from Noether’s theorem and gauge transformations [49]. By drawing inspiration from physical processes such as weak interactions, we can devise innovative generative models, such as ‘Yukawa generative models’ [50]. Viewing neural networks through the lens of physics can give us a deeper understanding of their structure and functionality from an entirely new perspective.

The synergy between AI and physics, two seemingly distinct fields, could lead to advancements in both domains. It is a two-fold benefit: AI could gain from the structured, universal laws of physics, and in return, physics could possibly leverage the predictive and analytical power of AI.

## CONCLUSION

This study reveals the remarkable link between quantum physics and neural networks, demonstrating that these artificial systems, like quantum systems, are subject to the uncertainty principle. This principle, often associated with precision and vulnerability trade-offs, provides new insights into the potential frailties inherent in neural networks.

Our findings also indicate that attacking the features of a neural network can be more effective than focusing on its pixels. This insight could possibly influence the optimization of network structures for better performance.

Meanwhile, viewing neural networks as complex physical systems allows us to apply principles from physics to understand the behaviour of these AI systems better. This interdisciplinary approach not only enhances our comprehension of AI systems, but also suggests a wealth of potential applications and advancements in both fields.

As we move forward, further exploration of this accuracy-robustness trade-off and its influence on the design of neural networks will be crucial. While this study provides a valuable perspective on the relationship between quantum physics and AI, additional research is still needed to more comprehensively understand how these principles can be applied to improve neural network robustness and design.

## METHODS

Detailed methods and materials are given in the online supplementary material.

## Supplementary Material

nwae141_Supplemental_File

## Data Availability

All data are available in the main text or the online supplementary material. Additional data related to this paper are available at https://doi.org/10.7910/DVN/SWDL1S and https://doi.org/10.48550/arXiv.2205.01493.
