# Supplementary material for: Quantum-inspired analysis of neural network vulnerabilities: the role of conjugate variables in system attacks
Source: Natl Sci Rev. 2024 Apr 11;11(9):nwae141. doi: 10.1093/nsr/nwae141 (PMC11321249; doi:10.1093/nsr/nwae141)
Supplement: nwae141_Supplemental_File [file nwae141_supplemental_file.pdf]

Quantum-Inspired Analysis of Neural Network Vulnerabilities: The Role of Conjugate Variables in System Attacks: Supplementary Material

Jun-Jie Zhang[1],Deyu Meng[2*]

[1]Division of Computational physics and Intelligent modeling,
Northwest Institute of Nuclear Technology,
Shaanxi, Xi'an 710024, China
E-mail: zjacob@mail.ustc.edu.cn
[2]School of Mathematics and Statistics,
Ministry of Education Key Lab of Intelligent Networks and Network Security
Xi'an Jiaotong University,
Shaanxi, P. R. China.

*To whom correspondence should be addressed; Email: dymeng@mail.xjtu.edu.cn

In this supplementary material, we provide the detailed definitions and proofs of the uncertainty principle proposed in the main text. Meanwhile, the numerical methods for estimating the high-dimensional integrals of the parameters are also presented.

1 Uncertainty Relation of the neural networks

1.1 Uncertainty principle in quantum physics

In quantum physics, we can describe a particle by a wave packet $\psi(X)$ in the coordinate representation with respect to the coordinate reference frame. The normalization condition

for $\psi(X)$ is given by

$$\int |\psi(X)|^2 dX \;=\; 1, \tag{1}$$

where the square amplitude $|\psi(X)|^2$ gives the probability density for finding a particle at position $X = (x, y, z)$. To measure the physical quantities of the particle, such as position X and momentum $P = (p_x, p_y, p_z)$, we need to define the position and momentum operators $\hat{x}_i$ and $\hat{p}_i$ as:

$$\begin{aligned} \hat{x}_i\psi(X) &= x_i\psi(X), \\ \hat{p}_i\psi(X) &= -i\frac{\partial}{\partial x_i}\psi(X), \end{aligned} \tag{2}$$

where $i = 1, 2, 3$ denote the x, y, z components in the coordinate space, respectively. The average position and momentum of the particle can be evaluated by

$$\begin{aligned} \langle \hat{x}_i \rangle &= \int \psi^*(X) x_i \psi(X) dX \\ \langle \hat{p}_i \rangle &= \int \psi^*(X)[-i\frac{\partial}{\partial x_i}\psi(X)]dX, \end{aligned} \tag{3}$$

where $\langle\cdot\rangle$ is the Dirac symbol widely used in physics and $\psi^*(X)$ is the complex conjugate of $\psi(X)$. The standard deviations of the position σ_{x_i} and momentum σ_{p_i} are defined respectively as:

$$\begin{aligned} \sigma_{x_i} &= \langle(\hat{x}_i - \langle\hat{x}_i\rangle)^2\rangle^{1/2}, \\ \sigma_{p_i} &= \langle(\hat{p}_i - \langle\hat{p}_i\rangle)^2\rangle^{1/2}. \end{aligned} \tag{4}$$

In the year of 1927, Heisenberg introduced the first formulation of the uncertainty principle in his German article[**?**]. The Heisenberg's uncertainty principle asserts a fundamental limit to the accuracy for certain pairs. Such variable pairs are known as complementary variables (or canonically conjugate variables). The formal inequality relating the standard

deviation of position σ_{x_i} and the standard deviation of momentum σ_{p_i} reads

$$\sigma_{x_i}\sigma_{p_i} \geq \frac{1}{2}. \tag{5}$$

Uncertainty relation Eq. (5) states a fundamental property of quantum systems and can be understood in terms of the Niels Bohr's complementarity principle[?]. That is, objects have certain pairs of complementary properties cannot be observed or measured simultaneously.

1.2 Formulas and notations for neural networks

Without loss of generality, we can assume that the loss function $l(f(X,\theta),Y)$ is square integrable[1],

$$\int l(f(X,\theta),Y)^2 dX = \beta. \tag{6}$$

Eq. (6) allows us to further normalize the loss function as

$$\psi_Y(X) = \frac{l(f(X,\theta),Y)}{\beta^{1/2}}, \tag{7}$$

so that

$$\int \psi_Y(X)^2 dX = 1. \tag{8}$$

For convenience, we refer $\psi_Y(X)$ as a neural packet in the later discussions. Note that under different labels Y, a neural network will be with a set of neural packets.

An image $X = (x_1, ..., x_i, ..., x_M)$ with M pixels can be seen as a point in the multi-dimensional space, where the numerical values of $(x_1, ..., x_i, ..., x_M)$ correspond to the pixel

[1] In practical applications, it is rational to only consider the loss function in a limited range $l(f(X,\theta),Y) < C$ under a large constant C, since samples out of this range can be seen as outliers and meaningless to the problem. The loss function can then be generally guaranteed to be square integrable in this functional range.

values. The feature and attack operators of the neural packet $\psi_Y(X)$ can then be defined as:

$$
\begin{aligned}
\hat{x}_i \psi_Y(X) &= x_i \psi_Y(X), \\
\hat{p}_i \psi_Y(X) &= \frac{\partial}{\partial x_i} \psi_Y(X).
\end{aligned} \tag{9}
$$

Similar as Eq. (3), the average pixel value at x_i associated with neural packet $\psi_Y(X)$ can be evaluated as

$$
\langle \hat{x}_i \rangle = \int \psi_Y^*(X) x_i \psi_Y(X)) dX. \tag{10}
$$

Since $\psi_Y(X)$ corresponds to a purely real number without imaginary part, the above equation is equivalent to:

$$
\langle \hat{x}_i \rangle = \int \psi_Y(X) x_i \psi_Y(X)) dX. \tag{11}
$$

Besides, the attack operator $\hat{p}_i = \frac{\partial}{\partial x_i}$ corresponds to the conjugate variable of x_i. And we can obtain the average value for $\hat{p}_i$ as

$$
\langle \hat{p}_i \rangle = \int \psi_Y(X) \frac{\partial}{\partial x_i} \psi_Y(X) dX. \tag{12}
$$

1.3 Derivation of the uncertainty relation

The uncertainty principle of a trained neural network can then be deduced by the following theorem:

> The standard deviations σ_{p_i} and σ_{x_i} corresponding to the attack and feature operators $\hat{p}_i$ and $\hat{x}_i$, respectively, are restricted by the relation:
>
> $$\sigma_{p_i} \sigma_{x_i} \geq \frac{1}{2}. \tag{13}$$

We first introduce the standard deviations σ_a and σ_b corresponding to two general operators $\hat{A}$ and $\hat{B}$. Then it follows that:

$$\sigma_a\sigma_b = \langle(\hat{A} - \langle\hat{A}\rangle)^2\rangle^{\frac{1}{2}}\langle(\hat{B} - \langle\hat{B}\rangle)^2\rangle^{\frac{1}{2}} \equiv \langle\hat{a}^2\rangle^{\frac{1}{2}}\langle\hat{b}^2\rangle^{\frac{1}{2}}. \tag{14}$$

In general, for any two unbounded real operators $\langle\hat{a}\rangle$ and $\langle\hat{b}\rangle$, the following relation holds

$$0 \leq \langle(\hat{a} - i\hat{b})^2\rangle = \langle\hat{a}^2\rangle - i\langle\hat{a}\hat{b} - \hat{b}\hat{a}\rangle + \langle\hat{b}^2\rangle. \tag{15}$$

If we further replace $\hat{a}$ and $\hat{b}$ in Eq. (15) by operators $\hat{a}\langle\hat{a}^2\rangle^{-1/2}$ and $\hat{b}\langle\hat{b}^2\rangle^{-1/2}$, we can then obtain the property $2\langle\hat{a}^2\rangle^{1/2}\langle\hat{b}^2\rangle^{1/2} \geq i\langle\hat{a}\hat{b} - \hat{b}\hat{a}\rangle$, which gives the basic bound for the commutator $[\hat{a}, \hat{b}] \equiv \hat{a}\hat{b} - \hat{b}\hat{a}$,

$$\langle\hat{a}^2\rangle^{\frac{1}{2}}\langle\hat{b}^2\rangle^{\frac{1}{2}} \geq |i\frac{1}{2}\langle[\hat{a}, \hat{b}]\rangle|. \tag{16}$$

Seeing the fact that $[\hat{a}, \hat{b}] = [\hat{A}, \hat{B}]$, we finally obtain the uncertainty relation

$$\sigma_a\sigma_b \geq |i\frac{1}{2}\langle[\hat{A}, \hat{B}]\rangle|. \tag{17}$$

In terms of the neural networks, we can simply replace operators $\hat{A}$ and $\hat{B}$ by $\hat{p}_i$ and $\hat{x}_i$ introduced in Eq. (9), and this leads to

$$\sigma_{p_i}\sigma_{x_i} \geq |i\frac{1}{2}\langle[\hat{p}_i, \hat{x}_i]\rangle| = \frac{1}{2}, \tag{18}$$

where we have used the relation

$$\begin{aligned}[\hat{p}_i, \hat{x}_i]\psi_Y(X) &= [\hat{p}_i\hat{x}_i - \hat{x}_i\hat{p}_i]\psi_Y(X) \\ &= \frac{\partial}{\partial x_i}[x_i\psi_Y(X)] \\ &\quad -x_i\frac{\partial}{\partial x_i}\psi_Y(X) \\ &= \psi_Y(X).\end{aligned} \tag{19}$$

Note that for a trained neural network, $\psi_Y(X)$ depends on the dataset and the structure of the network. Eq. (18) is a general result for general neural networks.

In the FGSM attack, the attacked image is of the form:

$$\begin{aligned} X &= X_0 + \epsilon \cdot sign(\nabla_X l(f(X,\theta), Y^*)|_{X=X_0}) \\ &\sim X_0 + \epsilon \cdot \nabla_X l(f(X,\theta), Y^*)|_{X=X_0} \\ &= X_0 + \epsilon \cdot \nabla_X [\beta^{1/2} \psi_{Y^*}(X_0)] \\ &= X_0 + \epsilon' \hat{P} \psi_{Y^*}(X_0), \end{aligned} \tag{20}$$

where $\hat{P} = (\frac{\partial}{\partial x_1}, ..., \frac{\partial}{\partial x_i}, ..., \frac{\partial}{\partial x_M})$ and $\epsilon' = \epsilon \cdot \beta^{1/2}$. In the second line of Eq. (20) we have used the property substantiated in [?]: ”even without the ’Sign’ of the FGSM, a successful attack can also be achieved". From Eq. (20), we can then obtain

$$\hat{P}\psi_{Y^*}(X_0) \sim \epsilon/\epsilon' \cdot sign(\nabla_X l(f(X,\theta), Y^*)|_{X=X_0}), \tag{21}$$

which is the reason that we call $\hat{p_i}$ the attack operator.

2 Evaluation of Δx and Δp

2.1 Approximation of Δx and Δp

In the equation referred to as Eq. (4), we encounter complex integrals involving σ_{x_i} and σ_{p_i}. These integrals are based on loss functions from trained neural networks and are challenging due to their high dimensionality. Specifically, they are 784-dimensional for the MNIST dataset and 3072-dimensional for the Cifar-10 dataset, which makes them impractical to calculate directly.

To work around this complexity, we simplify these multidimensional problems to a single dimension. Here’s how we do it using the MNIST dataset as an example:

We start by calculating the average value of all the input pixels, which we call X_{base}. Then, for a given trained classifier with a loss function, $l(f(X,\theta), Y = 8)$—where $Y = 8$ refers to the loss associated with the label number eight—we focus on one particular dimension, i, of the input X. We keep all other dimensions fixed at their base values, X_{base}. This reduces the loss function to depend on just one variable, x_i.

As a result, the complex equation (Eq. (6)) simplifies to the following one-dimensional integral:

$$\int l(f(X,\theta), Y = 8)^2 dX \Rightarrow \int l(f(x_i,\theta), Y = 8)^2 dx_i = \beta_i(Y = 8). \tag{22}$$

This integral can now be solved using the direct Monte-Carlo integration method. By repeating a similar process, we can calculate various quantities such as $\psi_{Y=8}(x_i)$, $\langle \hat{x}i(Y = 8)\rangle$, $\langle \hat{p}i(Y = 8)\rangle$, $\sigma_{x_i}(Y = 8)$, and $\sigma_{p_i}(Y = 8)$.

To get an overall estimate for label number eight, we randomly pick different i dimensions and then average them using the square-root of the sum:

$$\Delta X_8 \sim (\sum_i \sigma_{x_i}(Y = 8))^{1/2},\ \Delta P_8 \sim (\sum_i \sigma_{p_i}(Y = 8))^{1/2}. \tag{23}$$

This approach provides only an approximate estimate of the original high-dimensional integrals. While the results may not match the exact values, this estimation is useful as long as we are interested in the comparative trend of ΔX and ΔP, rather than their absolute values. Thus, this approximation is considered acceptable for our purposes.

2.2 Integral with Respect to Features and Pixels

In our research, we have employed three distinct neural network architectures. Upon completion of their training, these networks are partitioned into two segments: the feature

extractors and the classifiers, as illustrated in Fig. 1.

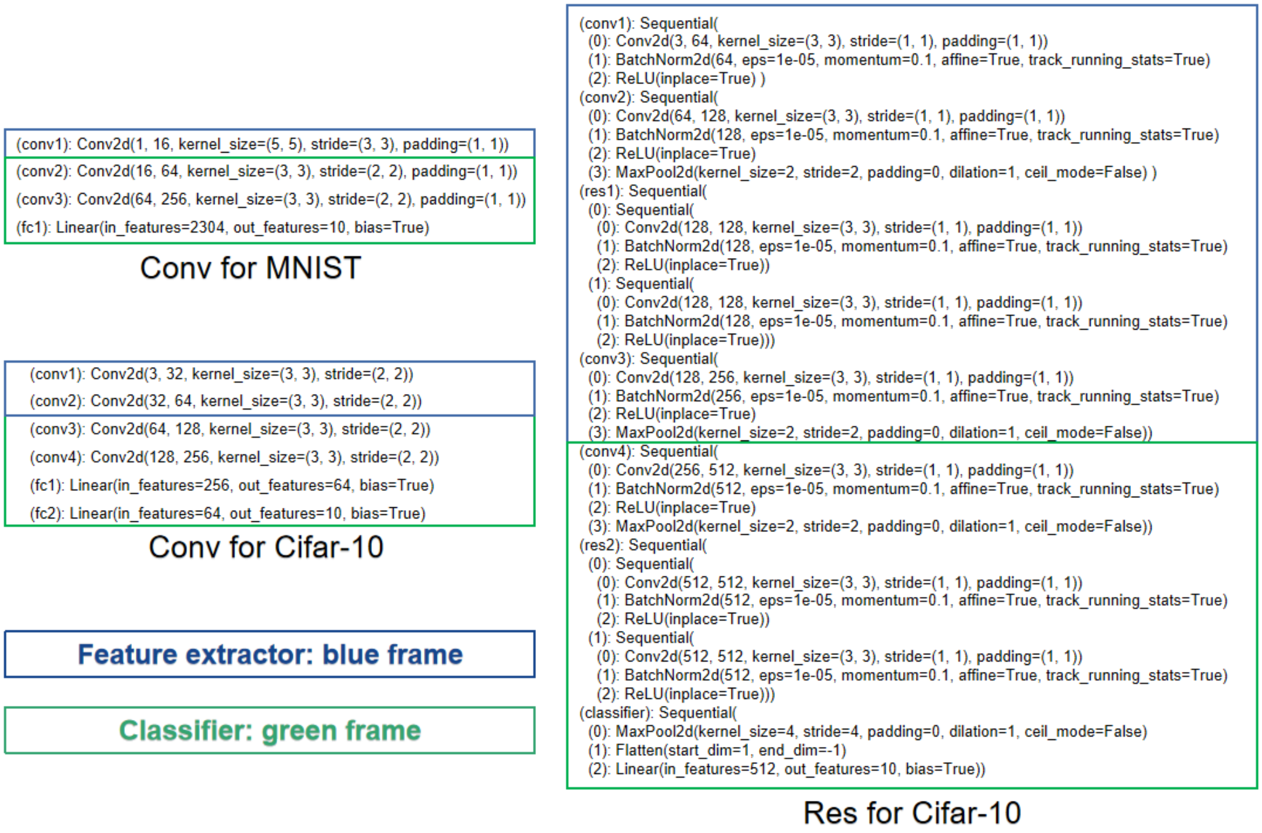

Figure 1: The three network structures used.

The integration process outlined in Equation (22) necessitates the use of an integrand space. This space can consist of either the unprocessed images at the pixel level or the attributes of the images that have undergone processing. Our study takes both scenarios into account in order to compare how the uncertainty principle manifests at each of these levels.

When performing the integration over pixel values, the loss functions associated with the three neural networks serve as the integrands and are evaluated using the Monte Carlo technique, effectively operating within the pixel space.

Alternatively, we initially process the images using the feature extractors, then we

retrain the classifiers with randomized weights to yield three refined classifiers. The loss functions related to these classifiers are then incorporated into Equation (22) to derive the values of ΔX and ΔP. In this instance, the integration is carried out over the space of extracted features.

References

[1] Heisenberg W. Über den anschaulichen inhalt der quantentheoretischen kinematik und mechanik. *Zeitschrift für Physik* 1927; **43**: 172–98.

[2] Bohr N. On the notions of causality and complementarity. *Science* 1950; **111**: 51–4.

[3] Agarwal A, Singh R and Vatsa M. The role of 'sign' and 'direction' of gradient on the performance of cnn Conference on Computer Vision and Pattern Recognition Workshops, Seattle: USA, 14-19 June 2020.
